# Differential gene expression reveals mechanisms related to habitat divergence between hybridizing orchids from the Neotropical coastal plains

**DOI:** 10.1186/s12870-020-02757-x

**Published:** 2020-12-10

**Authors:** Bárbara Simões Santos Leal, Marcelo Mendes Brandão, Clarisse Palma-Silva, Fabio Pinheiro

**Affiliations:** 1 Departamento de Biologia Vegetal, Universidade Estadual de Campinas, Campinas, SP 13083-862 Brazil; 2 Centro de Biologia Molecular e Engenharia Genética, Universidade Estadual de Campinas, Campinas, SP 13083-862 Brazil

**Keywords:** Ecological genomics, Edaphic adaptation, Neotropical speciation, Orchids, *Restingas*, Transcription profile

## Abstract

**Background:**

Closely related hybridizing species are ideal systems for identifying genomic regions underlying adaptive divergence. Although gene expression plays a central role in determining ecologically-based phenotypic differences, few studies have inferred the role of gene expression for adaptive divergence in Neotropical systems. In this study, we conduct genome-wide expression analysis alongside soil elemental analysis in sympatric and allopatric populations of *Epidendrum fulgens* and *E. puniceoluteum* (Orchidaceae), which occur in contrasting adjacent habitats in the Neotropical coastal plains.

**Results:**

These species were highly differentiated by their gene expression profiles, as determined by 18–21% of transcripts*.* Gene ontology (GO) terms associated with reproductive processes were enriched according to comparisons between species in both allopatric and sympatric populations. Species showed differential expression in genes linked to salt and waterlogging tolerance according to comparisons between species in sympatry, and biological processes related to environmental stimulus appeared as representative among those transcripts associated with edaphic characteristics in each sympatric zone. Hybrids, in their turn, were well differentiated from *E. fulgens,* but exhibited a similar gene expression profile to flooding-tolerant *E. puniceolutem.* When compared with parental species, hybrids showed no transcripts with additive pattern of expression and increased expression for almost all transgressive transcripts.

**Conclusions:**

This study sheds light on general mechanisms promoting ecological differentiation and assortative mating, and suggests candidate genes, such as those encoding catalase and calcium-dependent protein kinase, underling adaptation to harsh edaphic conditions in the Neotropical coastal plains. Moreover, it demonstrates that differential gene expression plays a central role in determining ecologically-based phenotypic differences among co-occurring species and their hybrids.

**Supplementary Information:**

The online version contains supplementary material available at 10.1186/s12870-020-02757-x.

## Background

Environmental heterogeneity is presumed to drive the adaptive differentiation of populations [1–3], ultimately leading to speciation [4, 5]. Speciation by polyploidy normally involves multiple ecological processes that are likely to strengthen the reproductive isolation resulting from post-mating mechanisms [6]. Ecological differentiation may aid the rising polyploid species to initially establish [6–8], thereby overcoming the frequency-dependent minority disadvantage in relation to diploid progenitors [9]. Studies have shown that polyploidy leads to increased number of genes and redundancy in function [10], and that new genetic variability have been a source for ecological novelties in polyploids [11], thus allowing their persistence in distinct niches from parental species [12, 13]. Polyploidy has also been commonly associated with adaptation to stressful environmental conditions unexplored by diploid species, particularly for plants [10].

Adaptation to edaphic habitats is deemed a major force underlying differential adaptation and speciation in plants (e.g., [14, 15]), including between species with distinct ploidy [16, 17]. Adaptive responses to soil conditions usually involve multiple phenotypic traits that are regulated by a number of small-effect loci largely influenced by genetic background and the environment [18, 19]. Studies on evolutionary ecology have increasingly employed high-throughput sequencing to investigate the genetic basis of edaphic adaptation at both the DNA sequence and gene expression levels [20]. Gene expression plays a central role in determining ecologically-based phenotypic differences and can unravel uneasily identifiable traits of ecological relevance [21, 22] such as physiological features. Therefore, the detection of gene expression divergence underlying ecologically important traits is considered a feasible starting point toward understanding the role of natural selection on the origin and maintenance of species [21]. Part of the genes whose expression is associated with adaptive divergence may also affect fitness, thereby contributing to ecologically-driven reproductive isolation [21, 23]. Although establishing these links is challenging due to the complex patterns of gene expression and scattered information on the genomic architecture for non-model organisms, differential gene expression can be used to generate further hypotheses about molecular mechanisms involved in reproductive isolation (e.g., [14, 24]).

**Fig. 1:**
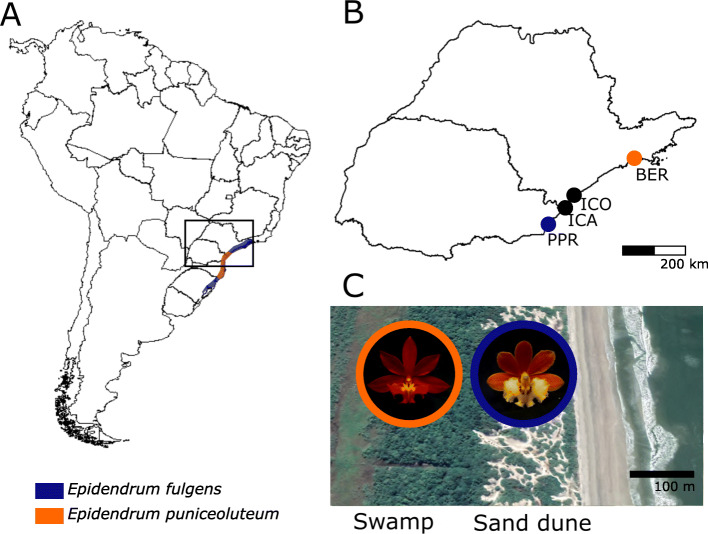
Distribution and sampling of *Epidendrum fulgens*, *E. puniceoluteum* and their hybrids. **a** Geographic distribution of *Epidendrum fulgens* and *E. puniceoluteum* in the Atlantic coastal areas. **b** Sampled populations in allopatry (blue for *E. fulgens*, orange for *E. puniceoluteum*) and sympatry (in black). BER = Bertioga - SP; ICO = Ilha Comprida - SP; ICA = Ilha do Cardoso - SP; PPR = Pontal do Paraná - PR. **c** Typical microhabitat in which each parental species occur: sand dunes for *E. fulgens*, sedge swamps for *E. puniceoluteum*. The aerial image was download from Google Earth Pro

Closely related hybridizing species are ideal systems for identifying genomic regions underlying adaptive divergence [25], particularly in species-rich regions (e.g., [26]). However, model systems are mostly from temperate regions of the northern hemisphere, limiting our understanding about the role of ecology on the process of adaptation and evolution of biodiversity in the tropics [27]. In our study, we investigate the genetic basis of adaptation in two closely related Neotropical orchids [28], the diploid *Epidendrum fulgens* and tetraploid *E. puniceoluteum*, which hybridize in the Atlantic coastal plains in southeast Brazil, a narrow vegetation physiognomy known as *restingas* [29, 30]. These species are associated with contrasting adjacent habitats; whereas *E. fulgens* predominantly occurs in sand dunes, *E. puniceoulutem* is found in swampy areas further inland (Fig. 1; Pinheiro et al. 2010). Thus, the extent to which soil factors, such as salinity, nutrients, and water availability, affect *E. fulgens* and *E. puniceoluteum* are likely to differ. Triploid hybrids, in their turn, are extensively distributed in both habitats [29–31] and might have a broader ecological niche than parental species as determined by transgressive patterns of gene inheritance [32, 33].

Up to date, few studies quantified genome-wide patterns of gene expression in ecologically divergent Neotropical species (but see [26]), and few empirical research has focused on the role of ecological divergence in polyploid speciation (but see [8]). Using RNA sequencing and soil composition data collected from sympatric and allopatric populations of *E. fulgens* and *E. puniceoluteum*, as well as their hybrids, we aimed at 1) comparing patterns of gene expression between parental species and identifying candidate genes putatively associated with adaptation to distinct soils; and 2) describing differential expressed genes that might explain the apparent broad habitat tolerance of hybrids when compared with parental species. Considering that differential expression is presumed to underlie the process of speciation [21], this approach provides some insights into general mechanisms involved in the origin and maintenance of high levels of biodiversity in Neotropical coastal plains.

## Results

### Edaphic niche differentiation

**Fig. 2:**
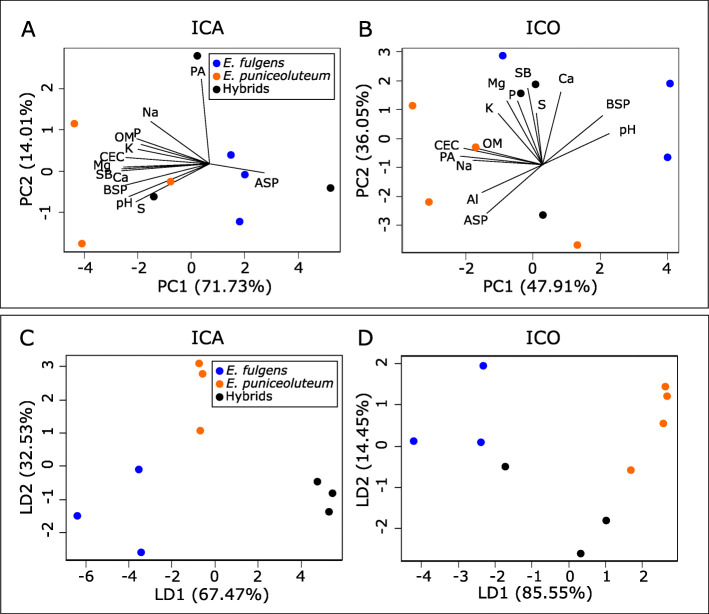
Principal Component Analysis (PCA) and Linear Discriminant Analysis (LDA) grouping soil variables from sites of *Epidendrum fulgens*, *E. puniceoluteum* and their hybrids. **a-b** Representation of the scores on the first two axes of the PCA in ICA and ICO hybrid zones, respectively, using soil variables measured in sites of *Epidendrum fulgens* (blue), *E. puniceoluteum* (orange) and their hybrids (black). Each arrow points in the direction of increase of values for the corresponding soil variable. pH = potential for hydrogen; OM = organic matter; CEC = cation exchange capacity; SB = sum of bases; BSP = base saturation percentage (BSP); ASP = aluminum saturation percentage; PA = potential acidity; P = phosphorus; K = potassium; Ca = calcium; Na = sodium; S = sulfur; Al = aluminum; Mg = magnesium. **c-d** Representation of the scores on the first two axes of the LDA in ICA and ICO hybrid zones, respectively, using the same soil variables

A Principal Component Analysis (PCA) evidenced slight edaphic differentiation between *E. puniceolutem* and *E. fulgens* along the PC1 for the ICA hybrid zone and along both PC1 and PC2 for the ICO hybrid zone (Fig. 2a-b). However, hybrids’ sites are not well differentiated from parental sites according to such analysis (Fig. 2a-b). The Linear Discriminant Analysis (LDA) shows that a combination of soil variables, particularly correlated to the concentrations of S and Ca (along LD1) and Na (along LD2, see Table S2), can properly differentiate *E. fulgens*, *E. puniceoluteum,* and hybrids’ sites from ICA (Fig. 2c). For ICO, in turn, the differentiation of *E. fulgens* and *E. puniceoluteum* occur along both axes of the PCA with contributions of all variables along either PC1 or PC2 (Fig. 2b). Conversely, LDA demonstrates that the discrimination between *E. fulgens* and *E. puniceouteum* sites in such hybrid zones is highly correlated with the variation in pH and K, which are variables mostly correlated to LD1 (Fig. 2d, Table S2). Nevertheless, soils associated with hybrids cannot be distinguished from those of parental species in ICO (Fig. 2).

### Reference transcriptome

We generated 9.53 × 10^7^ to 1.73 × 10^8^ paired-end raw reads per root-derived sample of *E. fulgens*, *E. puniceoluteum,* and hybrids (ca. 3.9 billion reads in total; raw reads are available at https://www.ncbi.nlm.nih.gov/sra/PRJNA678229), with PHRED score rates Q30 varying from 98.05 to 98.24% (Table S4). The final transcriptome resulted in a total of 247,978 contigs, with lengths varying from 200 to 15,424,225 bp (mean length = 832 bp). According to the BUSCO analysis, 1266 out of 1440 of the genes were complete (88%), while fragmented and missing genes corresponded to 4.7 and 7.3%, respectively. Blast2GO assigned gene ontology (GO) terms for 126,620 out of 202,425 contigs longer than 300 pb (63%).

### Gene expression patterns

**Fig. 3:**
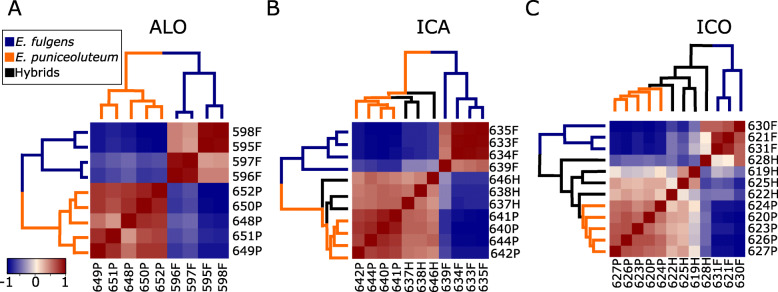
Patterns of differential gene expression among *Epidendrum fulgens*, *E. puniceoluteum* and their hybrids. Sample correlation matrix heatmaps for differentially expressed transcripts in comparisons between *E. fulgens* (in blue) and *E. puniceoluteum* (in orange) from allopatric populations (**a**), and among *E. fulgens, E. puniceoluteum* and hybrids (in black) from ICA (**b**) and ICO (**c**) sympatric zones

Gene expression analysis identified 19,062 differently expressed transcripts (DETs) between allopatric populations of each species (i.e., BER and PPR), from which 10,696 were upregulated in *E. puniceolutem* (Table 1). Comparisons between sympatric populations of *E. fulgens* and *E. puniceoluteum* indicated similar levels of differentiation, with 19,617 and 17,832 DETs within ICA and ICO, respectively. Slightly more DETs were upregulated in *E. puniceoluteum* in ICA and ICO (Table 1). These species are well differentiated by their expression profiles in sympatry and allopatry (Fig. 3a-c). A total of 11,689 DETs between *E. puniceoluteum* and *E. fulgens* commonly emerged from all comparisons, whereas 2419 DETs between these species were exclusively found in comparisons from sympatric zones.

**Table 1:** Number of differentially expressed transcripts (DETs) per pairwise comparison among *E. fulgens* (F), *E. puniceoluteum* (P) and hybrids (H) in allopatry and sympatry (ICO and ICA). The number of up-regulated and down-regulated transcripts refer to either *E. fulgens* (F) or *E. puniceoluteum* (P) in each comparison

Pairwise comparison	All transcripts	DETs	Up-regulated DETs	Down-regulated DETs
*E. fulgens* x *E. puniceoluteum* in allopatry	81,685	19,062 (23.34%)	8366(F)	10,696(F)
*E. fulgens* x *E. puniceoluteum* in simpatry (ICA)	63,562	19,617 (30.86%)	9187(F)	10,430(F)
*E. fulgens* x hybrids in simpatry (ICA)	66,940	16,707 (24.96%)	7749(F)	8958(F)
*E. puniceoluteum* x hybrids in simpatry (ICA)	76,582	1172 (1.53%)	1113(P)	59(P)
*E. fulgens* x *E. puniceoluteum* in simpatry (ICO)	80,348	17,832 (22.19%)	6691(F)	11,141(F)
*E. fulgens* x hybrids in simpatry (ICO)	109,784	9351 (8.52%)	47(F)	9304(F)
*E. puniceoluteum* x hybrids in simpatry (ICO)	120,138	2117 (1.76%)	2093(P)	24(P)

**Fig. 4:**
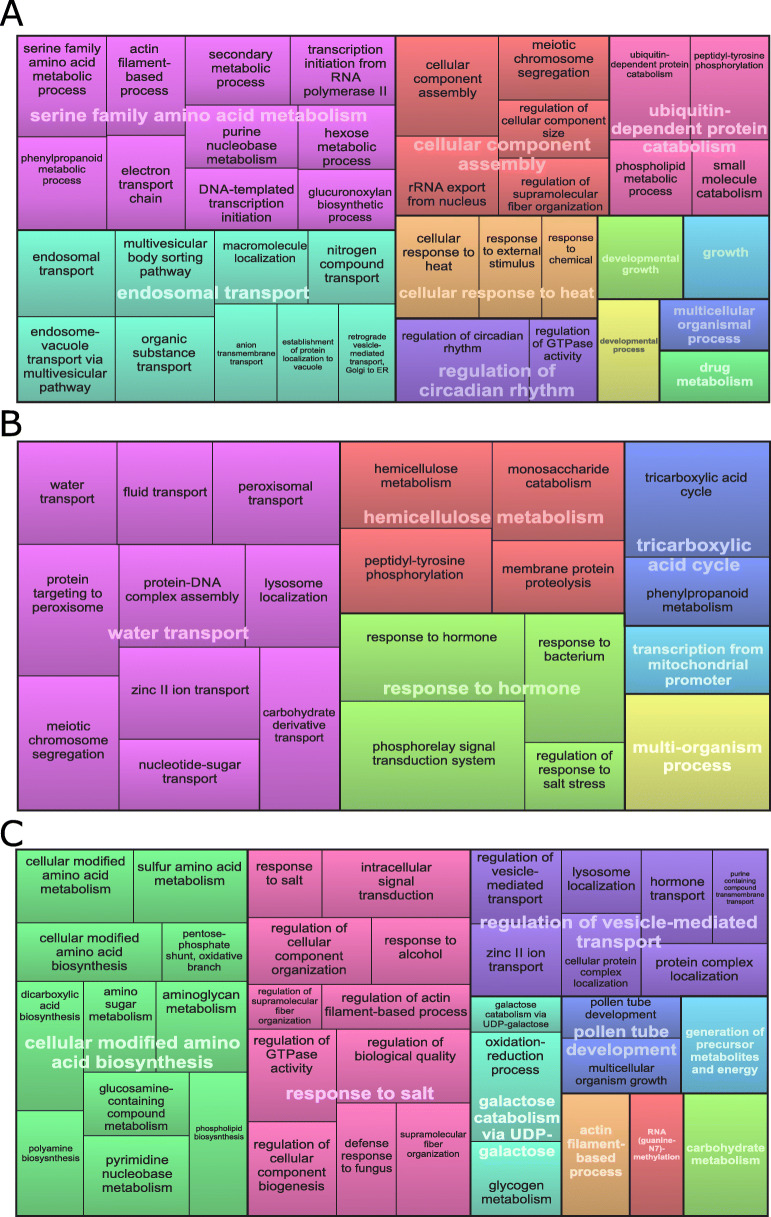
REVIGO treemaps summarizing representative gene ontology biological processes associated with enriched differentially expressed genes in comparisons between *E. fulgens* and *E. puniceoluteum*. **a** Results from comparisons between species in allopatry. **b-c** Results from comparisons between species in sympatry for ICA and ICO hybrid zones, respectively. Representatives terms are joined into superclusters of loosely related terms visualized with distinct colors. The size of the rectangles reflect *p*-values from enrichment analysis using topGO

Considering the gene ontology enrichment analysis, we identified 106 enriched GO terms between *E. fulgens* and *E. puniceoluteum* in allopatry, whereas 43 and 75 enriched GO terms were identified between species in the sympatric zones ICA and ICO, respectively. Two GO terms associated with chromosome segregation (GO:0045143 and GO:0045132) are commonly enriched in all comparisons. Three GO terms related to galactose catabolic process (GO:0033499), lysosome localization (GO:0032418), and zinc ion transport (GO:0006829) are over-represented only in comparisons between species in sympatry. Noteworthily, biological processes related to regulation of circadian rhythm (GO:0042752), growth (GO:0040007 and GO:0048589), and amino acid metabolism (GO:0009069) appear as representative GO terms for the analysis performed between species in allopatry (Table S5; Fig. 4). GO categories putatively associated with habitat differences between *E. fulgens* and *E. puniceoluteum* within each hybrid zone*,* such as water transport (GO:0006833), response to salt (GO:1902074), and regulation of response to salt stress (GO:1901000), phosphorelay signal transduction system (GO:0000160), and oxidation-reduction process (GO:0055114), are among the representative GO terms for comparisons within hybrid zones (see the treemaps in Fig. 4; Table S5).

**Fig. 5:**
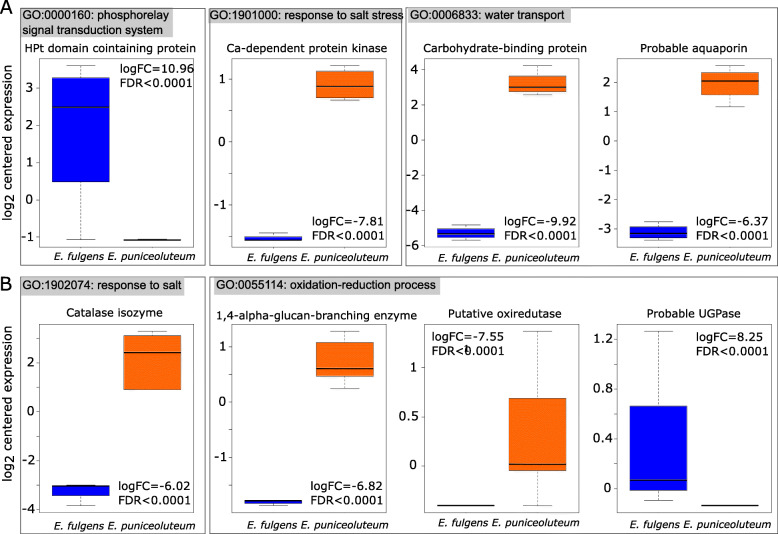
Expression levels of key transcripts underlying enriched gene ontology (GO) terms. (**a**) Gene expression differences between *Epidendrum ful*gens (in blue) and *E. puniceoluteum* (orange) from sympatric zones ICA and (**b**) ICO. Gene expression is shown as log_2_ transformed and centered values of FPKM. HPt = histidine-phosphotransferase; UGPase = UTP-glucose-1-phosphate uridylyltransferase. logFC = log_5_ fold change between species. FDR = False Discovery Rate (adjusted p-value)

Among DETs underlying enriched biological processes, we highlight those encoding the calcium-dependent protein kinase 26-like (CDK), catalase isozyme (CAT), histidine phosphotransfer protein (HPt), 1,4-α-glucan branching enzyme (GBE), glucose-1-phosphate uridylyltransferase (UGPase), and a putative oxireductase, which are likely involved in stress responses to waterlogging and salinity. These differentially expressed genes are upregulated in *E. puniceoluteum*, except for histidine phosphotransfer protein (HPt) and glucose-1-phosphate uridylyltransferase (UGPase) (Fig. 5a-b).

Within each hybrid zone, comparisons between *E. fulgens* and hybrids resulted in more DETs (Table 1) than comparisons between *E. puniceoluteum* and hybrids (Table 1). Indeed, hybrids are well differentiated from *E. fulgens,* but exhibit a similar gene expression profile to *E. puniceolutem* (Fig. 3b-c), with a higher proportion of transcripts exhibiting *E. puniceoluteum* dominance in hybrids (Table 2). We found no transcript showing additive patterns of inheritance in hybrids from the studied hybrid zones. We identified a total of 528 and 89 transcripts as transgressive in hybrids of ICA and ICO, respectively (Table 2). Most of these (i.e., 525 and 88, for ICA and ICO, respectively) are upregulated in hybrids when compared with both *E. fulgens* and *E. puniceoluteum*, although with high variance among sampled individuals (Fig. S1). When compared with conserved transcripts, transgressive transcripts have increased representation in biological processes related to response to stimulus, localization, and multi-organism and developmental processes in ICO (Fig. S2B).

**Table 2:** Number of transcripts showing conserved, additive, dominant, and transgressive gene expression patterns in hybrids from sympatric zones ICA and/or ICO

Pattern of inheritance in hybrids	ICA	ICO	ICA and ICO
Conserved	61,898	102,794	48,840
Dominant *E. puniceoluteum*	8509	9223	6252
Dominant *E. fulgens*	591	2010	126
Transgressive up-regulated	525	82	0
Transgressive down-regulated	3	2	0
Additive	0	0	0

### Gene-environment association

**Fig. 6:**
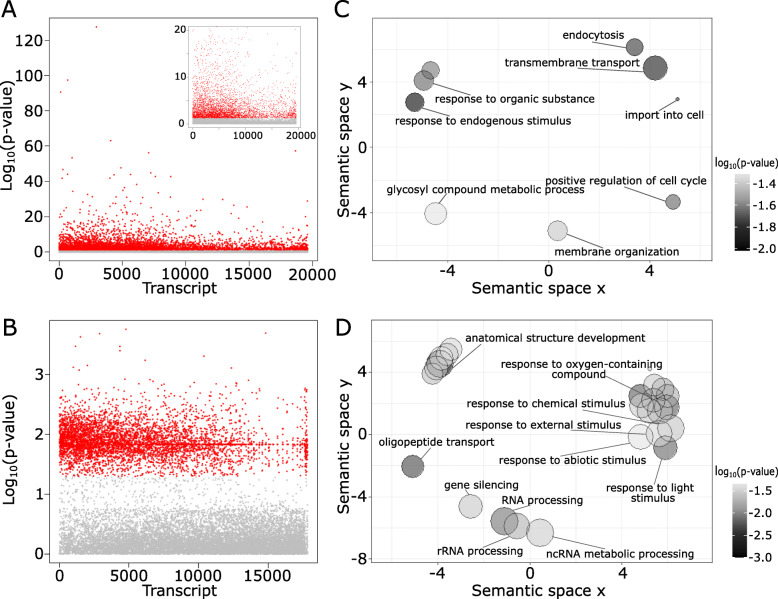
Latent Factor Mixed Models (LFMM) and similarity analysis for genes associated with edaphic variation in hybrid zones between *Epidendrum fulgens* and *E. puniceoluteum*. (**a**) Manhattan plot of 19,617 and 17,832 differently expressed transcripts (DETs) from ICA and (**b**) ICO hybrid zones, respectively. The plot represents minus log_10_ calibrated p-values obtained from LFMM. DETs with calibrated p-values < 0.05 (in red) are considered as significantly associated with the edaphic niche. (**c**) Semantic similarity analysis implemented in REVIGO showing representative (non-redundant) GO terms from enrichment analysis for DETs significantly associated to the edaphic niche of ICA and (**d**) ICO. The color of each bubble indicates the significance level of the GO term (darker bubbles have lower p-values in the enrichment analysis), while size indicates the frequency of the GO term in the UniProt database (bubbles of more general terms are larger)

Latent Factor Mixed Models (LFMMs) demonstrated a total of 4278 and 5018 DETs associated with the edaphic niche of ICA and ICO, respectively (Fig. 6a-b). According to the gene enrichment analysis, these transcripts are associated with 17 and 41 significant (*p* < 0.05) GO terms, respectively. For ICA, the semantic analysis resulted in a short list of biological processes related to responses to endogenous stimulus and organic substance (GO:0009719 and GO:0010033) as well as transmembrane transport (GO:0055085) and positive regulation of cell cycle (GO:0045787) (Fig. 6c). For ICO, in turn, several non-redundant GO terms associated with plant responses to abiotic conditions (GO:1901700, GO:0009416, GO:0042221, GO:0009628, and GO:0010035), and also with RNA processing (GO:0006396, GO:0006364 and GO:0034660) and anatomical development (GO:0048856), arose as representative in the semantic analysis (Fig. 6d).

## Discussion

Differential gene expression patterns in closely related species provide important insights into the role of ecology for differentiation and speciation. Gene expression profiles may uncover certain phenotypes that would not readily be visible via traditional approaches [5, 21], particularly those resulting from environmental gradients that impose multiple selective pressures and lead to complex physiological responses [34]. In our study, we show that the Neotropical hybridizing orchids *Epidendrum fulgens* and *E. puniceoluteum* from the Brazilian *restingas* are remarkably distinct at the gene expression level, with 18.16–23.59% of the genes being differentially expressed under natural conditions. Indeed, most differences in the species’ root transcripts are common to sympatric and allopatric comparisons and underlie general biological processes, which may correspond to species-specific constitutive responses. However, many of the gene expression differences seem to be linked to genetic mechanisms of adaptation and, hence, may provide information on factors contributing to the origin and/or maintenance of such species despite the ongoing interspecific gene flow [29]. These differences will be further discussed in light of ecological determinants likely affecting gene expression patterns in heterogeneous habitats within the Brazilian *restingas*.

According to discriminant analysis, edaphic sites of *E. fulgens* and *E. puniceoluteum* differ in pH, fertility, concentration of organic matter and sodium. In its turn, the gene expression analysis demonstrates that these species show acute differences in terms of adaptive responses to environmental stress. For instance, GO terms corresponding to processes related to stress response in plants, such as ‘water transport’, ‘regulation of response to salt stress’, ‘response to salt’, ‘phosphorelay signal transduction system’, and ‘oxidation-reduction process’, appear as representative in enrichment analyses between species within ICA and ICO sympatric zones. In addition, environment association analysis showed evidence of soil as predictor of gene expression in ca. 28 and 21% of DETs between *E. fulgens* and *E. puniceoluteum* from the two sympatric zones, respectively. Although we found no common enriched GO term associated with soils from both hybrid zones, these DETs are linked to several biological functions that reflect differential adaptation of *E. fulgens* and *E. puniceoluteum* to environmental conditions, particularly responses to stimulus, such as light, organic matter, and oxygen-containing compounds, and also anatomical development, membrane transport, and RNA processing. Specialization to distinct soils might, thus, have allowed the coexistence of closely related plant species within species-rich plant communities, such as the Brazilian *restingas*, where strong gradients of nutrients, salt, and water retention create highly variable edaphic conditions within small areas [35, 36].

Our results showed that genes underlying enriched biological processes, such as those encoding the calcium-dependent protein kinase 26-like (CDK) and the catalase isozyme (CAT), are upregulated in the tetraploid *E. puniceoluteum*. Such enzymes play multiple roles in response to various environmental stresses. While CDKs are involved in stress tolerance through the regulation of transcriptional activators or the translation of stress signals [37], CAT directly reacts with reactive oxygen species (ROS) keeping them at a low level [38]. The over expression of such enzymes in *E. puniceoluteum* may promote tolerance to salt stress, as have been demonstrated for crop and model species (rice: [39, 40]; *Arabidopsis* :[41]). On the other hand, the gene encoding the histidine phosphotransfer protein (HPt) appear to be downregulated in *E. puniceoluteum*. Indeed, an experimental study on the root tissues of *Aradapdopsis* showed downregulation of distinct HPt genes under osmotic stress conditions [42]. Such genes take part in one of the major intracellular signaling cascades, which is linked to several processes underlying survival under harsh conditions [43], such as osmosensing [44] and modulation of responses to salt stress (Pham [45]. Lastly, the expression of genes involved in oxidation-reduction processes – i.e., 1,4-α-glucan branching enzyme (GBE), glucose-1-phosphate uridylyltransferase (UGPase), and a putative oxireductase – also vary between *E. fulgens* and *E. puniceoluteum*. The synthesis of such enzymes is induced as a protection against ROS, which are accumulated in plants under both waterlogging and high salinity conditions creating oxidative stress [46–48]. Altogether, our results suggest a polygenic basis of multi-trait adaptation to distinct soil conditions and also a major role of stress-related genes in promoting divergence between species from *restingas*. Nevertheless, the process of linking specific genes to environment is not straightforward. Although this study indicates that species are edaphically specialized, further studies using an experimental approach would allow to disclose the role of such candidate genes in the putative differential fitness between species in each habitat (e.g., [49]).

In this study, we also showed that GO terms associated with reproduction and pollen tube development (i.e., ‘chromosome segregation’, ‘pollen tube development’, ‘cellular developmental process’, and ‘multicellular organism growth’) are over-represented in comparisons between species under both allopatric and sympatric conditions, which indicates a putative role of gene expression variation in the reproductive isolation between *E. fulgens* and *E. puniceoluteum*. Regulatory changes in genes involved in reproduction are predicted to underlie divergence during speciation, but few empirical research have actually shown that selection shape changes in their expression (see [24, 50]). Although links between genes whose expression is associated with adaptive divergence and genes involved in reproductive isolation (e.g., by pleiotropy, see [14]) cannot be established for this system yet, our results may be a feasible starting point toward understanding the role of selection in the origin and/or maintenance of *E. fulgens* and *E. puniceoluteum* despite the extensive interspecific gene flow [29].

The gene expression analysis we performed in this study demonstrated that the tetraploid *Epidendrum puniceoluteum* shares a similar root transcript profile with triploid hybrids, with a small proportion of transcripts differentially expressed in both sympatric zones (~ 1.5 and 1.7% in ICA and ICO, respectively). *Epidendrum puniceoluteum* expression dominance is also more frequent than *E. fulgens* dominance in hybrids from both sympatric zones. Indeed, hybrids are able to coexist with *E. puniceolutem* in swamp areas [29], probably overcoming the harmful and limiting effects of partial flooding for the establishment of plant species in *restingas* [36]. Root submergence leads to acute changes in the availability of oxygen, light, and nutrients, and triggers the inhibition of photosynthesis and the acceleration of energy reserve consumption [51], which has likely prevented the occurrence of *E. fulgens* in swamp areas. On the other hand, hybrids and *E. puniceoluteum* share a similar robust vegetative morphology (personal observation) and physiological adaptations that might confer tolerance to partial waterlogging. Such extensive ecological and morphological similarities may result from the unidirectional introgression toward the tetraploid *E. puniceoluteum* [29, 31], a recurrent pattern in diploid-tetraploid plant systems [52–54]. In fact, these hybrids have acted as triploid bridges for gene exchange between *E. fulgens* and *E. puniceoluteum* decreasing the genetic structure within populations [30, 31], and might also have played an additional role in the introgression across species boundaries, allowing for the adaptation to harsh edaphic conditions associated with flooding and other stressors.

The analysis of differential gene expression in hybrids compared with progenitors *E. fulgens* and *E. puniceoluteum* indicated that most DETs are upregulated in hybrids in relation to parental species in both hybrid zones, ICA and ICO. In addition, we found no additive patterns of expression and increased expression for almost all transgressive transcripts in hybrids. Such transgressive patterns of gene expression may result from a phenomenon called transcriptome shock [55, 56] or even from variation in gene dosage [57] considering that ploidy vary in this system. Changes of gene expression provide a source of physiological novelty in hybrids between *E. fulgens* and *E. puniceoluteum*. For ICO, the increased proportion of transgressive genes annotated to ‘developmental process’ and ‘multi-organism process’ suggest their key role in the divergence of hybrids from their parental species. However, most transgressive genes in hybrids are involved in cellular and metabolic processes, indicating that general processes may have enabled hybrids to occupy larger niches and could putatively lead them to survive in novel habitats not accessible to their parent species. Therefore, post-mating isolation due to reducing fitness of hybrids in parental habitats is not likely to represent a major reproductive barrier between *E. fulgens* and *E. puniceoluteum*. Rather, habitat divergence between these species may have acted as a premating barrier due to low viability of immigrants from parental species in the other’s habitat, thus promoting assortative mating in combination with post-mating isolation arising from meiotic failures (see [31]). In addition, since orchids depend on symbiotic fungus for germination and/or seedling development [58], an additional role of biotic interactions in promoting reproductive isolation between *E. fulgens* and *E. puniceoluteum* cannot be discarded.

## Conclusions

The role of gene expression for speciation is still a relatively unexplored topic, despite the increasing number of studies characterizing candidate genes or describing general patterns of gene expression related to adaptive divergence in different systems (e.g. [14, 24, 46]). Studies on gene expression patterns can simultaneously uncover variance in several ecophysiological traits, and thus help disentangling their adaptive importance for speciation. In this study we showed that differences in gene expression may have allowed the Neotropical orchids *E. fulgens* and *E. puniceolutrum* to coexist within small areas and can also be responsible for generating new phenotypic diversity in hybrids. Considering that we sampled material from natural populations, we cannot disregard the effect of other sources of variation on the observed gene expression profiles. However, we consider that our approach allows some candidate genes, i.e., putatively involved in ecological differences, to be tested for their relative importance in the speciation process by using physiological assays and/or gene-targeted approaches. It is not possible to affirm yet whether genes conferring adaptation to contrasting habitats took part of the initial reproductive isolation between *E. fulgens* and *E. puniceoluteum* or resulted from post-speciation adaptation, but divergent selection shall have played a critical role in maintaining divergence despite the high gene flow in this system. In future, we intend to test for alternative speciation scenarios for *E. fulgens* and *E. puniceoluteum* and the timing of divergence of adaptive genes in a model-based framework to address this issue.

## Methods

### Target species and plant sampling

*Epidendrum fulgens* inhabits the *restingas* vegetation along the coastal areas in southeastern and southern Brazil, in sand dunes not subject to flood and predominantly composed of shrubs (Fig. 1). *Epidendrum puniceoluteum* co-occurs with *E. fulgens* in a few localities along these coastal areas, and is generally found further inland (few meters apart) in sedge swamps (Fig. 1), i.e., depressions between successive beach ridges that are seasonally flooded (see [59]). Both species are pollinated by butterflies and produce no nectar, following a model of pollination by deceit that favor interspecific gene flow [28, 29, 60]. In sympatric areas, these species share pollinators and have overlapping flowering phenologies [29].

We sampled roots from adult individuals with similar size of *E. fulgens* and *E. puniceoluteum* and their hybrids in two hybrid zones, ICO and ICA, and in an allopatric population of each species BER and PPR (Fig. 1; Table S1). Sampled plants had 3–4 stems with 30–40 cm in length. Sampling was performed in April 2016 on sunny days, between 13:00 and 17:00, with temperatures ranging from 27 °C to 30 °C. Root samples were collected from flowering individuals and immediately stored in RNAlater™ Stabilization Solution (Thermo Fisher Scientific). All samples were further kept under − 80 °C until RNA extraction. We collected plants under natural conditions to ensure assessment of patterns of gene expression as close as possible to natural populations, which are likely influenced by a combination of innate and environment-dependent mechanisms. The identity of each sampled individual samples were a priori determined by F. Pinheiro (senior author) based on morphological characteristics and further confirmed using a Bayesian assignment analysis with microsatellites markers and flow cytometry (see Appendix 1 for a detailed description of the employed methods and obtained results). Vouchers of each species and population are deposited in the Herbarium of the Instituto de Botânica de São Paulo (SP), São Paulo, Brazil (see Table S1). The collection of plant material complied with national guidelines. Permits to collect in conservation units were granted by COTEC (Technical and Scientific Committee of the Forestry Institute, São Paulo, Brazil; permission number 279/2017) and SISBIO (Biodiversity Information and Authorization System, Brazil, permission number 15206–3).

### Soil characterization

To characterize soil conditions, we sampled root-proximal soil (~ 0.5 kg from the surface layer) associated with individuals of *E. fulgens, E. puniceoluteu* and their hybrids from sympatric zones ICA and ICO for multi-element analysis. We determined the soil pH, organic matter (OM), cation exchange capacity (CEC), sum of bases (SB), base saturation percentage (BSP), aluminum saturation percentage (ASP), potential acidity (PA), as well as extractable and exchangeable concentrations of phosphorus (P), potassium (K), calcium (Ca), sodium (Na), sulfur (S), aluminum (Al), and magnesium (Mg) in soil samples as described by [61]. The variation in these multiple soil variables within each hybrid zone were summarized using a principal component analysis (PCA) implemented in R software. Then, we performed a linear discriminant analysis (LDA) using the R-package MASS [62] to identify variables that best explain differences among the three predefined groups (i.e., soil sites of *E. fulgens*, *E. puniceoluteum,* and their hybrids).

### RNA extraction, library preparation, and sequencing

We extract RNA from roots using the Agilent Plant RNA Isolation Mini Kit (Agilent Technologies Inc.; Santa Clara, CA, USA) and sent the RNA samples to Macrogen Inc. (Seoul, Korea). cDNA libraries were constructed using the TruSeq RNA Sample Prep Kit v.2. A total of 32 samples were individually barcoded and paired-end sequenced on a single lane of the Illumina NovaSeq™ 6000 system (San Diego, CA, USA) following the manufacturer’s protocol.

### Transcriptome assembly and annotation

A “hybrid transcriptome” was de novo assembled to be used as a reference for the gene expression analysis and, therefore, to avoid bias toward one of the parental species. To do so, we merged reads from all sampled hybrids into a single dataset, and filtered low-quality bases (Phred score < 30) and adapters using the Fastx-toolkit (http://hannonlab.cshl.edu/fastx_toolkit). Filtered data were normalized using the “normalize_by_kmer_coverage” procedure of the Trinity v. 2.6.6 pipeline [63]. Subsequently, a de novo assembly was performed using the MIRA assembler v. 4.9.4 [64] under the following settings: quality clipping on (−CL:qc = yes), spoiler detection on (−AS:sd = yes), minimum base quality = 5 (−CL:qcmq = 5), length of the window for quality clipping = 5 (−CL:qcwl = 5); relative percentage of exact word matches = 70% (−SK:pr = 70, Stepping increment = 2 (−SK:kss = 2); maximum mega-hub ratio = 1 (−SK:mmhr = 1), and minimum number of reads to discard sequences = 3 (−AS:mrpc = 3). To avoid chimeric contigs, we discarded reads mapped in more than one contig or mapped more than once within the same contig using the blast_check tool. This reference transcriptome was filtered for contigs longer than 200 bp with a minimum of 5x coverage. We assessed the quality and completeness of this assembled transcriptome using the BUSCO tool [65] with the Embryophyta odb9 dataset.

We used Blast2GO [66] to perform a functional annotation of the reference transcriptome. We filtered for contigs longer than 300 bp and then aligned the contigs against the NCBI non-redundant NR protein database with an e-value cut-off of 10e− 3 using the CloudBlast service. Simultaneously, we used the InterProScan tool to retrieve protein domains and motif information. Lastly, we processed all matching transcripts and assigned gene ontology (GO) terms following the Blast2GO annotation workflow.

### Analyses of gene expression and gene ontology enrichment

Pairwise differential expression analyses were carried out between *E. fulgens* and *E. puniceoluteum* allopatric populations (BER and PPR) and among *E. fulgens*, *E. puniceoluteum,* and their hybrids within each hybrid zone (ICO and ICA) following the Trinity pipeline. To do so, we mapped reads to the reference transcriptome using Bowtie 2 implemented in the RSEM 2.01 software [67] to summarize read counts of each library; then, performed the differential gene expression analysis based on TMM normalized FPKM values using edgeR package [68] with a false discovery rate (FDR) cut-off of 0.001 and at least 2^5^ fold expression differentiation.

We performed a GO enrichment analysis to identify over-represented biological processes among differentially expressed transcripts (DETs) using the Elim–Kolmogorov–Smirnov method with a significance threshold of 0.05 as implemented in the R topGO package [69]. This method allowed testing for enriched GO terms while accounting for the topology of the GO graph (conditional enrichment). To summarize information coming from enriched GO terms and exclude redundant terms (similarity > 0.5), we used a semantic analysis implemented in the REVIGO Web server [70]. Genes underlying known biological processes associated with edaphic adaptation were further extracted, and their expression was compared between species in each sympatric zone. We also extract commonly enriched GO terms between parental species in comparisons under sympatric (within ICA and ICO) and allopatric (between PPR and BER) conditions, as well as GO terms exclusively shared by both sympatric zones (ICA and ICO), in order to reveal general patterns of ecological differentiation. Lastly, we extracted and summarized GO terms associated with transcripts showing conservative, transgressive, additive, or dominant patterns of inheritance in hybrids from ICA and ICO sympatric zones using the WEGO tool [71]. Within each hybrid zone, a transcript was considered to be conservative if similarly expressed in hybrids*, E. fulgens,* and *E. puniceoluteum*; and transgressive when hybrids expression was significantly higher or lower than both parental species, as detected by the edgeR package. Differentially expressed transcripts whose expression in the hybrid was higher than one of the parents but lower than another, were considered additive. Finally, inheritance was considered dominant if the expression of hybrids was significantly different from one parental species but not from the other.

### Environment-driven gene expression

To obtain a set of DETs exhibiting significant correlations with the edaphic niche, we performed a latent factor mixed model (LFMM) implemented in the R package ‘lfmm’ [72]. The ecological association test implemented in ‘lfmm’ accounts for unobserved confounding effects and extends the typical definition of response matrix from genotypic to gene expression data [72]. The analysis was separately carried out for each sampled hybrid zone (i.e., ICA and ICO). To account for putative confounders, the number of latent factors (K) was a priori defined through a PCA as K = 2 for both ICA and ICO hybrid zones. The TMM normalized FPKM values for each DET were used as response variables, and the first principal component from a PCA of 14 soil variables was used as the predictor variable. The first component accounts for 71% of the variance among ICA samples and is moderately correlated to all soil variables with the exception of PA (Table S3). For ICO, in its turn, the first component accounts for 47.7% of the total variance and is moderately associated with pH, organic matter, concentration of Al and Na, as well as CEC, BSP, and ASP (Table S3). We used the ridge approach implemented in ‘lfmm’ package to minimize the least-squares problem based on L2 penalty. The list of significant *p*-values (< 0.05) was obtained by using the genomic inflation method, which controls for the false discovery rate.

A GO enrichment analysis was further performed to identify over-represented biological processes among DETs associated with the edaphic niche. To do so, we used the Elim–Kolmogorov–Smirnov method with a significance threshold of 0.05 as implemented in the R topGO package. Lastly, we summarized information coming from the enriched GO terms and excluded redundant terms (similarity > 0.5), using the semantic analysis implemented in the REVIGO Web server.

## Supplementary Information

**Additional file 1: Figure S1.** Heatmaps showing the expression of 528 and 89 transgressive genes in hybrids relative to parental species *Epidendrum fulgens* and *E. puniceoluteum* in ICA (A) and ICO (B) hybrid zones. Each row represents a single transcript and the colors from light gray to black denote expression levels (log_2_ transformed centered values of FPKM). Transgressive transcripts are up regulated in hybrids, with exception of three down regulated transcripts in ICA and a single down-regulated transcript in ICO (marked with arrows). **Figure S2.** Categorized gene ontology (GO) terms associated with conserved, additive, dominant (either *Epidendrum fulgens*, F, or *E. puniceoluteum*, P), and transgressive modes of inheritance in hybrids from sympatric zones ICA (A) and ICO (B). GO terms are summarized into molecular functions and biological process categories according to WEGO. **Table S1.** Number of sampled individuals (N) of *E. fulgens*, *E. puniceolutem*, and hybrids for each sympatric and allopatric population. Vouchers for each species and location are deposited in the Herbarium of the Instituto de Botânica de São Paulo (SP), Brazil. **Table S2.** Coefficients of linear discriminants of a LDA using soil data from sites of *Epidendrum fulgens*, *E. puniceoluteum* and their hybrids in ICA and ICO. Values hilighted in bold correspond to variables more correlated to the first and second linear discriminant axis (LD1 and LD2, respectively). **Table S3.** Correlations between soil variables and the first principal component (PC1) of PCAs using soil data for sites of *Epidendrum fulgens*, *E. puniceoluteum* and their hybrids in ICA and ICO. **Table S4**. RNA sequencing summary information for individuals of *Epidendrum fulgens*, *E. puniceoluteum*, and hybrids. GC = guanine-cytosine content; PHRED scores Q20 = accuracy of a base call of 99% and Q30 = accuracy of a base call of 99.9%. **Table S5.** Non-redundant gene ontology enriched terms (*p* < 0.05) for differentially expressed genes between *E. fulgens* and *E. puniceoluteum* in allopatry (BER vs PPR) and sympatry (within ICO and ICA) using the Elim–Kolmogorov–Smirnov method implemented in TopGO.

**Additional file 2: Appendix 1.** Samples identity according to a Bayesian assignment analysis with microsatellites markers and flow cytometry.

## Data Availability

Raw sequencing have been deposited in the NCBI SRA database (https://www.ncbi.nlm.nih.gov/sra/PRJNA678229; BioProject ID: PRJNA678229; BioSample accessions: SAMN16790585-SAMN16790616). The assembled transcriptome and gene annotation are available in the FigShare repository [10.6084/m9.figshare.c.5203004.v1].

## Abbreviations

CAT: Catalase isozyme
CDK: Calcium-dependent protein kinase 26-like
DET: Differently expressed transcript
GBE: 1,4-α-glucan branching enzyme
GO: Gene ontology
HPt: Histidine phosphotransfer protein
ROS: Reactive oxygen species
UGPase: Glucose-1-phosphate uridylyltransferase

## References

[1] Lowry DB, Hall MC, Salt DE, Willis JH (2009). Genetic and physiological basis of adaptive salt tolerance divergence between coastal and inland *Mimulus guttatus*. New Phytol.

[2] Wang GD, Zhang BL, Zhou WW, Li YX, Jin JQ, Shao Y (2018). Selection and environmental adaptation along a path to speciation in the Tibetan frog *Nanorana parkeri*. Proc Natl Acad Sci.

[3] Savolainen O, Lascoux M, Merilä J (2013). Ecological genomics of local adaptation. Nat Rev Genet.

[4] Feder JL, Egan SP, Nosil P (2012). The genomics of speciation-with-gene-flow. Trends Genet.

[5] Nosil P (2012). Ecological speciation.

[6] Sobel JM, Chen GF, Watt LR, Schemske DW (2010). The biology of speciation. Evolution.

[7] Husband BC, Sabara HA (2004). Reproductive isolation between autotetraploids and their diploid progenitors in fireweed, *Chamerion angustifolium* (Onagraceae). New Phytol.

[8] Pegoraro L, Cafasso D, Rinaldi R, Cozzolino S, Scopece G (2016). Habitat preference and flowering-time variation contribute to reproductive isolation between diploid and autotetraploid *Anacamptis pyramidalis*. J Evol Biol.

[9] Levin DA (2002). The Role of Chromosomal Change in Plant Evolution. Oxford Series in Ecology and Evolution.

[10] Alix K, Gérard PR, Schwarzacher T, Heslop-Harrison JS (2017). Polyploidy and interspecific hybridization: partners for adaptation, speciation and evolution in plants. Ann Bot.

[11] Ramsey J, Schemske DW (2002). Neopolyploidy in flowering plants. Annu Rev Ecol Systematics.

[12] Soltis PS, Soltis DE (2000). The role of genetic and genomic attributes in the success of polyploids. Proc Natl Acad Sci.

[13] Wei N, Cronn R, Liston A, Ashman TL (2019). Functional trait divergence and trait plasticity confer polyploid advantage in heterogeneous environments. New Phytol.

[14] Dunning LT, Hipperson H, Baker WJ, Butlin RK, Devaux C, Hutton I (2016). Ecological speciation in sympatric palms: 1. Gene expression, selection and pleiotropy. J Evol Biol.

[15] Melo MC, Grealy A, Brittain B, Walter GM, Ortiz-Barrientos D (2014). Strong extrinsic reproductive isolation between parapatric populations of an Australian groundsel. New Phytol.

[16] Shimizu-Inatsugi R, Terada A, Hirose K, Kudoh H, Sese J, Shimizu KK (2017). Plant adaptive radiation mediated by polyploid plasticity in transcriptomes. Mol Ecol.

[17] Han TS, Zheng QJ, Onstein RE, Rojas-Andrés BM, Hauenschild F, Muellner-Riehl AN, Xing YW (2020). Polyploidy promotes species diversification of *Allium* through ecological shifts. New Phytol.

[18] Turner TL, Bourne EC, Von Wettberg EJ, Hu TT, Nuzhdin SV (2010). Population resequencing reveals local adaptation of *Arabidopsis lyrata* to serpentine soils. Nat Genet.

[19] Wang X, Chen ZH, Yang C, Zhang X, Jin G, Chen G, Dai F (2018). Genomic adaptation to drought in wild barley is driven by edaphic natural selection at the Tabigha evolution slope. Proc Natl Acad Sci.

[20] Rajakaruna N (2018). Lessons on evolution from the study of edaphic specialization. Bot Rev.

[21] Pavey SA, Collin H, Nosil P, Rogers SM (2010). The role of gene expression in ecological speciation. Ann N Y Acad Sci.

[22] Alvarez M, Schrey AW, Richards CL (2015). Ten years of transcriptomics in wild populations: what have we learned about their ecology and evolution?. Mol Ecol.

[23] Nosil P, Vines TH, Funk DJ (2005). Reproductive isolation caused by natural selection against immigrants from divergent habitats. Evolution..

[24] Tusso LE, S. Caminade P, Severac D, Boursot P, Ganem G, Smadja CM. (2017). Do changes in gene expression contribute to sexual isolation and reinforcement in the house mouse?. Mol Ecol.

[25] Payseur BA, Rieseberg LH (2016). A genomic perspective on hybridization and speciation. Mol Ecol.

[26] Kelley JL, Arias-Rodriguez L, Martin DP, Yee MC, Bustamante CD, Tobler M (2016). Mechanisms underlying adaptation to life in hydrogen sulfide-rich environments. Mol Biol Evol.

[27] Beheregaray LB, Cooke GM, Chao NL, Landguth EL (2015). Ecological speciation in the tropics: insights from comparative genetic studies in Amazonia. Front Genet.

[28] Cardoso-Gustavson P, Saka MN, Pessoa EM, Palma-Silva C, Pinheiro F (2018). Unidirectional transitions in nectar gain and loss suggest food deception is a stable evolutionary strategy in *Epidendrum* (Orchidaceae): insights from anatomical and molecular evidence. BMC Plant Biol.

[29] Pinheiro F, de Barros F, Palma-Silva C, Meyer D, Fay MF, Suzuki RM (2010). Hybridization and introgression across different ploidy levels in the Neotropical orchids *Epidendrum fulgens* and *E. puniceoluteum* (Orchidaceae). Mol Ecol.

[30] Sujii PS, Cozzolino S, Pinheiro F (2019). Hybridization and geographic distribution shapes the spatial genetic structure of two co-occurring orchid species. Heredity..

[31] Moraes AP, Chinaglia M, Palma-Silva C, Pinheiro F (2013). Interploidy hybridization in sympatric zones: the formation of *Epidendrum fulgens* × *E. puniceoluteum* hybrids (Epidendroideae, Orchidaceae). Ecol Evol.

[32] Rieseberg LH, Archer MA, Wayne RK (1999). Transgressive segregation, adaptation and speciation. Heredity..

[33] Arnold ML, Martin NH (2010). Hybrid fitness across time and habitats. Trends Ecol Evol.

[34] Sobel JM, Stankowski S, Streisfeld MA (2019). Variation in ecophysiological traits might contribute to ecogeographic isolation and divergence between parapatric ecotypes of *Mimulus aurantiacus*. J Evol Biol.

[35] Scarano FR (2002). Structure, function and floristic relationships of plant communities in stressful habitats marginal to the Brazilian Atlantic rainforest. Ann Bot.

[36] Magnago LF, Martins SV, Schaefer CE, Neri AV (2012). Restinga forests of the Brazilian coast: richness and abundance of tree species on different soils. An Acad Bras Cienc.

[37] Tang W, Luo C (2018). Overexpression of zinc finger transcription factor ZAT6 enhances salt tolerance. Open Life Sci.

[38] Smirnof N (1993). The role of active oxygen in the response of plants to water deficit and desiccation. New Phytol.

[39] Shim IS, Momose Y, Yamamoto A, Kim DW, Usui K (2003). Inhibition of catalase activity by oxidative stress and its relationship to salicylic acid accumulation in plants. Plant Growth Regul.

[40] Wei S, Hu W, Deng X, Zhang Y, Liu X, Zhao X (2014). A rice calcium dependent protein kinase OsCPK9 positively regulates drought stress tolerance and spikelet fertility. BMC Plant Biol.

[41] Tang W, Page M (2013). Overexpression of the *Arabidopsis* AtEm6 gene enhances salt tolerance in transgenic rice cell lines. Plant Cell Tissue Organ Culture.

[42] Singh A, Kushwaha HR, Soni P, Gupta H, Singla-Pareek SL, Pareek A (2015). Tissue specific and abiotic stress regulated transcription of histidine kinases in plants is also influenced by diurnal rhythm. Front Plant Sci.

[43] Sharan A, Soni P, Nongpiur RC, Singla-Pareek SL, Pareek A (2017). Mapping the ‘two-component system’network in rice. Sci Rep.

[44] Urao T, Yakubov B, Satoh R, Yamaguchi-Shinozaki K, Seki M, Hirayama T, Shinozaki K (1999). A transmembrane hybrid-type histidine kinase in *Arabidopsis* functions as an osmosensor. Plant Cell.

[45] Pham J, Liu J, Bennett MH, Mansfield JW, Desikan R (2012). *Arabidopsis* histidine kinase 5 regulates salt sensitivity and resistance against bacterial and fungal infection. New Phytol.

[46] Blokhina O, Virolainen E, Fagerstedt KV (2003). Antioxidants, oxidative damage and oxygen deprivation stress: a review. Ann Bot.

[47] Peng Y, Zhou Z, Zhang Z, Yu X, Zhang X, Du K (2018). Molecular and physiological responses in roots of two full-sib poplars uncover mechanisms that contribute to differences in partial submergence tolerance. Sci Rep.

[48] Van Breusegem F, Vranová E, Dat JF, Inzé D (2001). The role of active oxygen species in plant signal transduction. Plant Sci.

[49] Li A, Li L, Wang W, Zhang G (2019). Evolutionary trade-offs between baseline and plastic gene expression in two congeneric oyster species. Biol Lett.

[50] Chapman MA, Hiscock SJ, Filatov DA (2013). Genomic divergence during speciation driven by adaptation to altitude. Mol Biol Evol.

[51] Fukao T, Xiong L (2013). Genetic mechanisms conferring adaptation to submergence and drought in rice: simple or complex?. Curr Opin Plant Biol.

[52] Ståhlberg D, Hedrén M (2010). Evolutionary history of the *Dactylorhiza maculata* polyploid complex (Orchidaceae). Biol J Linn Soc.

[53] Chapman MA, Abbott RJ (2010). Introgression of fitness genes across a ploidy barrier. New Phytol.

[54] Zohren J, Wang N, Kardailsky I, Borrell JS, Joecker A, Nichols RA, Buggs RJ (2016). Unidirectional diploid–tetraploid introgression among British birch trees with shifting ranges shown by restriction site-associated markers. Mol Ecol.

[55] Han H, Sun X, Xie Y, Feng J, Zhang S (2014). Transcriptome and proteome profiling of adventitious root development in hybrid larch (*Larix kaempferi*× *Larix olgensis*). BMC Plant Biol.

[56] Wu Y, Sun Y, Wang X, Lin X, Sun S, Shen K (2016). Transcriptome shock in an interspecific F1 triploid hybrid of *Oryza* revealed by RNA sequencing. J Integr Plant Biol.

[57] Riddle NC, Birchler JA (2003). Effects of reunited diverged regulatory hierarchies in allopolyploids and species hybrids. Trends Genet.

[58] Rasmussen HN (2002). Recent developments in the study of orchid mycorrhiza. Plant Soil.

[59] Araujo DD. Vegetation types of sandy coastal plains of tropical Brazil: a first approximation. In Coastal plant communities of Latin America (pp. 337–347). Cambridge: Academic Press; 1999.

[60] Moreira ASFP, Fuhro D, dos Santos Isaias RM (2008). Anatomia floral de *Epidendrum fulgens* Brongn.(Orchidaceae-Epidendroideae) com ênfase no nectário e sua funcionalidade. Rev Biol Neotrop J Neotrop Biol.

[61] Embrapa. Manual de análises químicas de solo, planta e fertilizantes. 2nd edition. Brasilia:Embrapa Informações Tecnologicas; 2009.

[62] Venables WN, Ripley BD. Modern applied statistics with S, Fourth edition. New York: Springer; 2002.

[63] Haas BJ, Papanicolaou A, Yassour M, Grabherr M, Blood PD, Bowden J (2013). De novo transcript sequence reconstruction from RNA-seq using the trinity platform for reference generation and analysis. Nat Protoc.

[64] Chevreux B, Pfisterer T, Drescher B, Driesel AJ, Müller WE, Wetter T, Suhai S (2004). Using the miraEST assembler for reliable and automated mRNA transcript assembly and SNP detection in sequenced ESTs. Genome Res.

[65] Seppey M, Manni M, Zdobnov EM, Kollmar M (2019). BUSCO: assessing genome assembly and annotation completeness. Gene prediction. Methods in molecular biology, vol 1962.

[66] Götz S, García-Gómez JM, Terol J, Williams TD, Nagaraj SH, Nueda MJ, Conesa A (2008). High-throughput functional annotation and data mining with the Blast2GO suite. Nucleic Acids Res.

[67] Li B, Dewey CN. RSEM: accurate transcript quantification from RNA-Seq data with or without a reference genome. BMC Bioinformatics. 2011;12:23. 10.1186/1471-2105-12-323 PMC3163565 21816040

[68] Robinson MD, McCarthy DJ, Smyth GK (2010). edgeR: a bioconductor package for differential expression analysis of digital gene expression data. Bioinformatics..

[69] Alexa A, Rahnenfuhrer J. topGO: Enrichment Analysis for Gene Ontology. R package version 2.42.0, 2020.

[70] Supek F, Bošnjak M, Škunca N, Šmuck T (2011). REVIGO summarizes and visualizes long lists of gene ontology terms. PLoS One.

[71] Ye J, Zhang Y, Cui H, Liu J, Wu Y, Cheng Y (2018). WEGO 2.0: a web tool for analyzing and plotting GO annotations, 2018 update. Nucleic Acids Res.

[72] Caye K, Jumentier B, Lepeule J, François O (2019). LFMM 2: fast and accurate inference of gene-environment associations in genome-wide studies. Mol Biol Evol.

