# Supplementary material for: Differential gene expression reveals mechanisms related to habitat divergence between hybridizing orchids from the Neotropical coastal plains
Source: BMC Plant Biol. 2020 Dec 10;20:554. doi: 10.1186/s12870-020-02757-x (PMC7731501; doi:10.1186/s12870-020-02757-x)
Supplement: Supplementary file 1 — Additional file 1: Figure S1. Heatmaps showing the expression of 528 and 89 transgressive genes in hybrids relative to parental species Epidendrum fulgens and E. puniceoluteum in ICA (A) and ICO (B) hybrid zones. Each row represents a single transcript and the colors from light gray to black denote expression levels (log2 transformed centered values of FPKM). Transgressive transcripts are up regulated in hybrids, with exception of three down regulated transcripts in ICA and a single down-regulated transcript in ICO (marked with arrows). Figure S2. Categorized gene ontology (GO) terms associated with conserved, additive, dominant (either Epidendrum fulgens, F, or E. puniceoluteum, P), and transgressive modes of inheritance in hybrids from sympatric zones ICA (A) and ICO (B). GO terms are summarized into molecular functions and biological process categories according to WEGO. Table S1. Number of sampled individuals (N) of E. fulgens, E. puniceolutem, and hybrids for each sympatric and allopatric population. Vouchers for each species and location are deposited in the Herbarium of the Instituto de Botânica de São Paulo (SP), Brazil. Table S2. Coefficients of linear discriminants of a LDA using soil data from sites of Epidendrum fulgens, E. puniceoluteum and their hybrids in ICA and ICO. Values hilighted in bold correspond to variables more correlated to the first and second linear discriminant axis (LD1 and LD2, respectively). Table S3. Correlations between soil variables and the first principal component (PC1) of PCAs using soil data for sites of Epidendrum fulgens, E. puniceoluteum and their hybrids in ICA and ICO. Table S4. RNA sequencing summary information for individuals of Epidendrum fulgens, E. puniceoluteum, and hybrids. GC = guanine-cytosine content; PHRED scores Q20 = accuracy of a base call of 99% and Q30 = accuracy of a base call of 99.9%. Table S5. Non-redundant gene ontology enriched terms (p < 0.05) for differentially expressed genes between E. fulgens a [file 12870_2020_2757_MOESM1_ESM.docx]

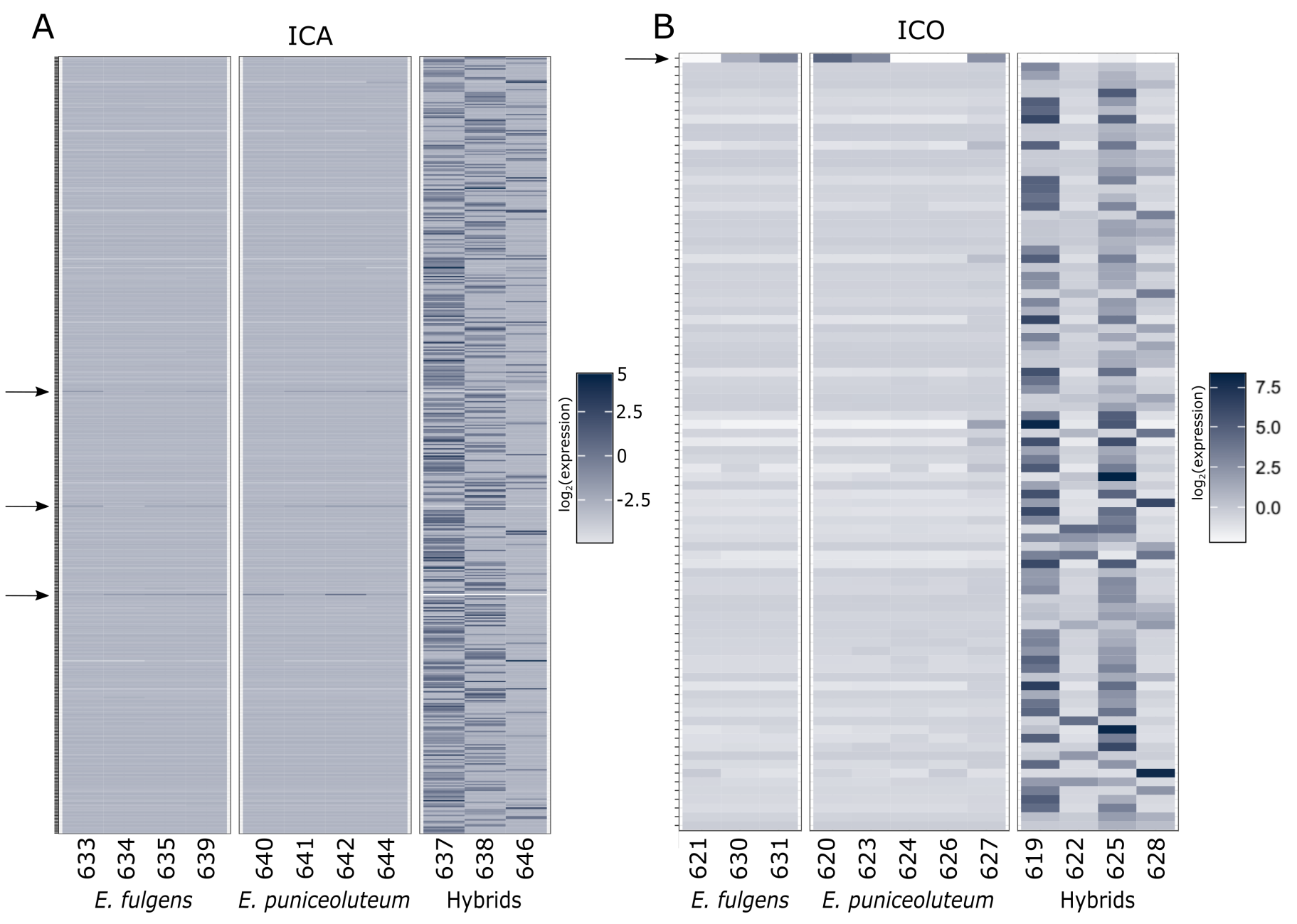


**Figure S1.** Heatmaps showing the expression of 528 and 89 transgressive genes in hybrids relative to parental species *Epidendrum fulgens* and *E. puniceoluteum* in ICA (A) and ICO (B) hybrid zones. Each row represents a single transcript and the colors from light gray to black denote expression levels (log_2_ transformed centered values of FPKM). Transgressive transcripts are up regulated in hybrids, with exception of three down regulated transcripts in ICA and a single down-regulated transcript in ICO (marked with arrows).


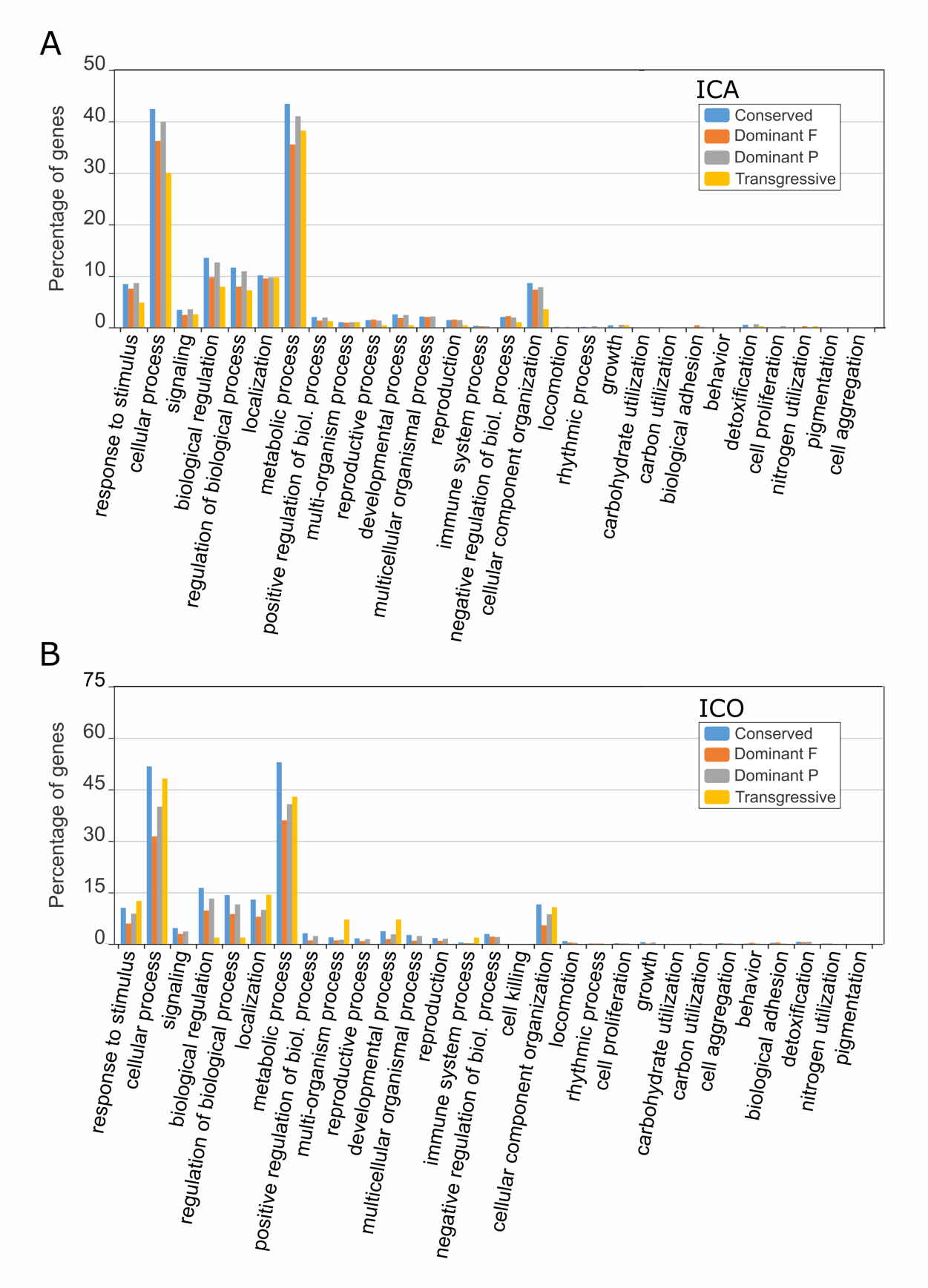


**Figure S2.** Categorized gene ontology (GO) terms associated with conserved, additive, dominant (either *Epidendrum fulgens*, F, or *E. puniceoluteum*, P), and transgressive modes of inheritance in hybrids from sympatric zones ICA (A) and ICO (B). GO terms are summarized into molecular functions and biological process categories according to WEGO.

**Table S1.** Number of sampled individuals (N) of *E. fulgens*, *E. puniceolutem*, and hybrids for each sympatric and allopatric population. Vouchers for each species and location are deposited in the Herbarium of the Instituto de Botânica de São Paulo (SP), Brazil.

| **Id** | **Species** | **Locality** | **Coordinates (lat-long)** | | **Voucher** | **N_RNA_** | **N_SOIL_** |
| --- | --- | --- | --- | --- | --- | --- | --- |
| ICA | *E. fulgens* | Ilha do Cardoso - SP | -25.0657 | -47.9096 | F. Pinheiro 632 | 4 | 3 |
|  | *E. puniceoluteum* |  |  |  | F. Pinheiro 529 | 4 | 3 |
|  | Hybrids |  |  |  | F. Pinheiro 655 | 3 | 3 |
| ICO | *E. fulgens* | Ilha Comprida - SP | -24.8583 | -47.7087 | F. Pinheiro 653 | 3 | 3 |
|  | *E. puniceoluteum* |  |  |  | F. Pinheiro 528 | 5 | 4 |
|  | Hybrids |  |  |  | F. Pinheiro 654 | 4 | 3 |
| BER | *E. fulgens* | Bertioga - SP | -23.7786 | -45.9590 | F. Pinheiro 616 | 4 | - |
| PPR | *E. puniceoluteum* | Pontal do Paraná - PR | -25.6332 | -48.4259 | F. Pinheiro 621 | 5 | - |
| Total | | | | | | 32 | 19 |

**Table S2.** Coefficients of linear discriminants of a LDA using soil data from sites of *Epidendrum fulgens*, *E. puniceoluteum* and their hybrids in ICA and ICO. Values hilighted in bold correspond to variables more correlated to the first and second linear discriminant axis (LD1 and LD2, respectively).

| **Soil variable** | **ICA** | | **ICO** | |
| --- | --- | --- | --- | --- |
|  | **LD1** | **LD2** | **LD1** | **LD2** |
| pH | -3.068 | -0.856 | **-0.740** | 0.193 |
| Organic matter (OM) | 1.424 | 0.926 | -0.082 | 0.231 |
| Phosphorus (P) | -0.268 | 0.660 | 0.193 | **-0.947** |
| Sulfur (S) | **5.326** | -0.030 | -0.534 | **-1.207** |
| Potassium (K) | 3.000 | -0.491 | **0.786** | -0.038 |
| Calcium (Ca) | **-3.418** | 0.897 | -0.377 | -0.432 |
| Magnesium (Mg) | 1.537 | 0.798 | -0.059 | **0.911** |
| Aluminium (Al) |  |  | 0.113 | -0.272 |
| Sodium (Na) | -0.829 | **1.592** | -0.084 | -0.107 |
| Potential acidity (PA) | 0.278 | -0.978 | 0.115 | 0.550 |
| Sum of bases (SB) | -0.800 | 0.236 | -0.118 | 0.231 |
| Cation Exchange Capacity (CEC) | 0.850 | -0.960 | -0.001 | 0.336 |
| % Base Saturation (BSP) | -2.504 | 0.182 | -0.307 | 0.782 |
| % Aluminium Saturation (ASP) | 2.920 | 1.040 | **0.786** | -0.171 |

**Table S3.** Correlations between soil variables and the first principal component (PC1) of PCAs using soil data for sites of *Epidendrum fulgens*, *E. puniceoluteum* and their hybrids in ICA and ICO.

| **Soil variable** | **ICA** | **ICO** |
| --- | --- | --- |
| pH | -0.300 | 0.334 |
| Organic matter (OM) | -0.290 | -0.356 |
| Phosphorus (P) | -0.276 | -0.119 |
| Sulfur (S) | -0.258 | -0.033 |
| Potassium (K) | -0.270 | -0.216 |
| Calcium (Ca) | -0.325 | 0.093 |
| Magnesium (Mg) | -0.326 | -0.170 |
| Aluminium (Al) | - | -0.294 |
| Sodium (Na) | -0.223 | -0.350 |
| Potential acidity (PA) | -0.035 | -0.383 |
| Sum of bases (SB) | -0.325 | -0.068 |
| Cation Exchange Capacity (CEC) | -0.316 | -0.373 |
| % Base Saturation (BSP) | -0.310 | 0.301 |
| % Aluminium Saturation (ASP) | 0.212 | -0.268 |

**Table S4**. RNA sequencing summary information for individuals of *Epidendrum fulgens*, *E. puniceoluteum*, and hybrids. GC = guanine-cytosine content; PHRED scores Q20 = accuracy of a base call of 99% and Q30 = accuracy of a base call of 99.9%.

| **Id** | **Species** | **Locality** | **Base pairs** | **Raw reads** | **GC (%)** | **Q20 (%)** | **Q30 (%)** |
| --- | --- | --- | --- | --- | --- | --- | --- |
| 595F | *E. fulgens* | BER | 1.48E+10 | 146,116,152 | 49.9 | 98.95 | 98.14 |
| 596F | *E. fulgens* | BER | 1.39E+10 | 137,645,954 | 49.37 | 98.99 | 98.22 |
| 597F | *E. fulgens* | BER | 1.30E+10 | 129,124,264 | 49.6 | 98.94 | 98.12 |
| 598F | *E. fulgens* | BER | 1.07E+10 | 105,734,802 | 49.51 | 98.92 | 98.06 |
| 619H | Hybrid | ICO | 1.38E+10 | 136,939,166 | 49.39 | 99.01 | 98.24 |
| 620P | *E. puniceoluteum* | ICO | 1.33E+10 | 131,329,984 | 49.44 | 98.99 | 98.2 |
| 621F | *E. fulgens* | ICO | 1.27E+10 | 125,888,854 | 49.38 | 99.01 | 98.23 |
| 622H | Hybrid | ICO | 1.28E+10 | 126,419,460 | 49.15 | 98.99 | 98.22 |
| 623P | *E. puniceoluteum* | ICO | 1.32E+10 | 130,744,672 | 49.21 | 98.97 | 98.16 |
| 624P | *E. puniceoluteum* | ICO | 1.49E+10 | 147,797,680 | 49.52 | 99.01 | 98.24 |
| 625H | Hybrid | ICO | 1.25E+10 | 123,478,038 | 49.35 | 98.93 | 98.07 |
| 626P | *E. puniceoluteum* | ICO | 1.31E+10 | 129,997,472 | 49.06 | 98.95 | 98.13 |
| 627P | *E. puniceoluteum* | ICO | 1.21E+10 | 119,369,566 | 49.26 | 98.99 | 98.18 |
| 628H | Hybrid | ICO | 1.33E+10 | 131,196,416 | 49.32 | 98.92 | 98.07 |
| 630F | *E. fulgens* | ICO | 1.04E+10 | 103,119,498 | 49.65 | 98.96 | 98.13 |
| 631F | *E. fulgens* | ICO | 1.17E+10 | 115,462,574 | 49.3 | 98.95 | 98.13 |
| 633F | *E. fulgens* | ICA | 1.22E+10 | 121,038,270 | 49.63 | 99.01 | 98.24 |
| 634F | *E. fulgens* | ICA | 1.14E+10 | 112,483,290 | 49.45 | 98.95 | 98.13 |
| 635F | *E. fulgens* | ICA | 1.43E+10 | 141,949,580 | 49.21 | 98.96 | 98.16 |
| 637H | Hybrid | ICA | 1.05E+10 | 104,082,852 | 49.62 | 98.99 | 98.22 |
| 638H | Hybrid | ICA | 9.63E+09 | 95,330,530 | 49.43 | 98.98 | 98.2 |
| 639F | *E. fulgens* | ICA | 1.75E+10 | 172,908,938 | 49.7 | 98.99 | 98.21 |
| 640P | *E. puniceoluteum* | ICA | 1.45E+10 | 143,842,706 | 49.21 | 98.96 | 98.17 |
| 641P | *E. puniceoluteum* | ICA | 1.03E+10 | 102,204,004 | 49.07 | 98.97 | 98.2 |
| 642P | *E. puniceoluteum* | ICA | 1.05E+10 | 103,731,374 | 48.87 | 98.99 | 98.22 |
| 644P | *E. puniceoluteum* | ICA | 1.07E+10 | 105,737,024 | 48.79 | 98.96 | 98.16 |
| 646H | Hybrid | ICA | 1.04E+10 | 103,294,862 | 48.56 | 98.93 | 98.1 |
| 648P | *E. puniceoluteum* | PPR | 1.06E+10 | 104,942,102 | 49.16 | 98.98 | 98.21 |
| 649P | *E. puniceoluteum* | PPR | 1.06E+10 | 105,158,226 | 48.57 | 98.89 | 98.05 |
| 650P | *E. puniceoluteum* | PPR | 1.06E+10 | 105,301,432 | 49.06 | 98.96 | 98.15 |
| 651P | *E. puniceoluteum* | PPR | 1.30E+10 | 128,328,942 | 48.92 | 98.98 | 98.18 |
| 652P | *E. puniceoluteum* | PPR | 1.05E+10 | 103,597,358 | 48.37 | 99 | 98.24 |
| **Total** | | | 3.93E+11 | 3,894,296,042 | - | - | - |

**Table S5.** Non-redundant gene ontology enriched terms (p<0.05) for differentially expressed genes between *E. fulgens* and *E. puniceoluteum* in allopatry (BER vs PPR) and sympatry (within ICO and ICA) using the Elim–Kolmogorov–Smirnov method implemented in TopGO.

| **GO_term** | **Description** | **Annotated** | **Significant** | **Expected** | **p(elimKS)** |
| --- | --- | --- | --- | --- | --- |
| ***E. fulgens* vs *E. puniceoluteum* in allopatry** | | | | | |
| GO:0006144 | purine nucleobase metabolic process | 3 | 0 | 0.65 | 0.0230 |
| GO:0006352 | DNA-templated transcription, initiation | 8 | 1 | 1.73 | 0.0231 |
| GO:0006367 | transcription initiation from RNA polymerase II promoter | 5 | 1 | 1.08 | 0.0192 |
| GO:0006407 | rRNA export from nucleus | 4 | 0 | 0.86 | 0.0119 |
| GO:0006644 | phospholipid metabolic process | 56 | 12 | 12.09 | 0.0351 |
| GO:0006890 | retrograde vesicle-mediated transport, Golgi to ER | 13 | 2 | 2.81 | 0.0479 |
| GO:0009069 | serine family amino acid metabolic process | 11 | 3 | 2.37 | **0.0071** |
| GO:0009605 | response to external stimulus | 57 | 7 | 12.31 | 0.0311 |
| GO:0009698 | phenylpropanoid metabolic process | 22 | 1 | 4.75 | 0.0108 |
| GO:0010417 | glucuronoxylan biosynthetic process | 9 | 0 | 1.94 | 0.0409 |
| GO:0016197 | endosomal transport | 16 | 4 | 3.45 | 0.0104 |
| GO:0017144 | drug metabolic process | 105 | 28 | 22.67 | 0.0476 |
| GO:0018108 | peptidyl-tyrosine phosphorylation | 91 | 6 | 19.65 | 0.0117 |
| GO:0019318 | hexose metabolic process | 32 | 11 | 6.91 | 0.0359 |
| GO:0019748 | secondary metabolic process | 37 | 4 | 7.99 | 0.0191 |
| GO:0022607 | cellular component assembly | 119 | 23 | 25.69 | **0.0038** |
| GO:0022900 | electron transport chain | 9 | 1 | 1.94 | 0.0183 |
| GO:0030029 | actin filament-based process | 11 | 1 | 2.37 | 0.0142 |
| GO:0032501 | multicellular organismal process | 123 | 16 | 26.55 | 0.0474 |
| GO:0032502 | developmental process | 159 | 20 | 34.33 | 0.0337 |
| GO:0032511 | late endosome to vacuole transport via multivesicular body sorting pathway | 16 | 4 | 3.45 | 0.0104 |
| GO:0032535 | regulation of cellular component size | 8 | 1 | 1.73 | 0.0327 |
| GO:0033036 | macromolecule localization | 262 | 59 | 56.56 | 0.0252 |
| GO:0034605 | cellular response to heat | 13 | 3 | 2.81 | 0.0106 |
| GO:0040007 | growth | 28 | 2 | 6.04 | 0.0220 |
| GO:0042221 | response to chemical | 138 | 19 | 29.79 | 0.0463 |
| GO:0042752 | regulation of circadian rhythm | 20 | 1 | 4.32 | **0.0025** |
| GO:0043087 | regulation of GTPase activity | 5 | 1 | 1.08 | 0.0458 |
| GO:0043162 | ubiquitin-dependent protein catabolic process via the multivesicular body sorting pathway | 16 | 4 | 3.45 | 0.0104 |
| GO:0044282 | small molecule catabolic process | 66 | 16 | 14.25 | 0.0421 |
| GO:0045132 | meiotic chromosome segregation | 6 | 0 | 1.3 | 0.0214 |
| GO:0048589 | developmental growth | 28 | 2 | 6.04 | 0.0220 |
| GO:0071702 | organic substance transport | 327 | 73 | 70.59 | 0.0105 |
| GO:0071705 | nitrogen compound transport | 313 | 69 | 67.57 | 0.0313 |
| GO:0071985 | multivesicular body sorting pathway | 16 | 4 | 3.45 | 0.0104 |
| GO:0072666 | establishment of protein localization to vacuole | 22 | 5 | 4.75 | 0.0407 |
| GO:0098656 | anion transmembrane transport | 10 | 2 | 2.16 | 0.0361 |
| GO:1902903 | regulation of supramolecular fiber organization | 8 | 1 | 1.73 | 0.0327 |
| ***E. fulgens* vs *E. puniceoluteum* in sympatry (ICA)** | | | | | |
| GO:0000160 | phosphorelay signal transduction system | 22 | 2 | 4.38 | 0.0173 |
| GO:0006099 | tricarboxylic acid cycle | 8 | 0 | 1.59 | 0.0025 |
| GO:0006390 | transcription from mitochondrial promoter | 8 | 0 | 1.59 | 0.0145 |
| GO:0006625 | protein targeting to peroxisome | 4 | 0 | 0.8 | 0.0209 |
| GO:0006829 | zinc II ion transport | 2 | 0 | 0.4 | 0.0464 |
| GO:0006833 | water transport | 12 | 2 | 2.39 | 0.0162 |
| GO:0009617 | response to bacterium | 4 | 0 | 0.8 | 0.0194 |
| GO:0009698 | phenylpropanoid metabolic process | 22 | 1 | 4.38 | 0.0255 |
| GO:0009725 | response to hormone | 74 | 10 | 14.73 | 0.0046 |
| GO:0010410 | hemicellulose metabolic process | 43 | 4 | 8.56 | 0.0090 |
| GO:0015780 | nucleotide-sugar transport | 6 | 0 | 1.19 | 0.0492 |
| GO:0018108 | peptidyl-tyrosine phosphorylation | 91 | 9 | 18.11 | 0.0129 |
| GO:0032418 | lysosome localization | 3 | 0 | 0.6 | 0.0349 |
| GO:0033619 | membrane protein proteolysis | 15 | 1 | 2.99 | 0.0177 |
| GO:0042044 | fluid transport | 12 | 2 | 2.39 | 0.0162 |
| GO:0043574 | peroxisomal transport | 4 | 0 | 0.8 | 0.0209 |
| GO:0045132 | meiotic chromosome segregation | 7 | 0 | 1.39 | 0.0281 |
| GO:0046365 | monosaccharide catabolic process | 5 | 0 | 1 | 0.0132 |
| GO:0051704 | multi-organism process | 48 | 5 | 9.55 | 0.0430 |
| GO:0065004 | protein-DNA complex assembly | 11 | 1 | 2.19 | 0.0292 |
| GO:1901000 | regulation of response to salt stress | 8 | 1 | 1.59 | 0.0396 |
| GO:1901264 | carbohydrate derivative transport | 6 | 0 | 1.19 | 0.0492 |
| ***E. fulgens* vs *E. puniceoluteum* in sympatry (ICO)** | | | | | |
| GO:0000096 | sulfur amino acid metabolic process | 55 | 6 | 7.83 | 0.0063 |
| GO:0005975 | carbohydrate metabolic process | 618 | 92 | 88.03 | 0.0477 |
| GO:0005977 | glycogen metabolic process | 35 | 5 | 4.99 | 0.0176 |
| GO:0006022 | aminoglycan metabolic process | 16 | 2 | 2.28 | 0.0243 |
| GO:0006040 | amino sugar metabolic process | 16 | 2 | 2.28 | 0.0243 |
| GO:0006091 | generation of precursor metabolites and energy | 82 | 10 | 11.68 | 0.0159 |
| GO:0006206 | pyrimidine nucleobase metabolic process | 11 | 0 | 1.57 | 0.0390 |
| GO:0006575 | cellular modified amino acid metabolic process | 17 | 1 | 2.42 | 0.0008 |
| GO:0006596 | polyamine biosynthetic process | 4 | 0 | 0.57 | 0.0161 |
| GO:0006829 | zinc II ion transport | 2 | 0 | 0.28 | 0.0254 |
| GO:0008654 | phospholipid biosynthetic process | 49 | 5 | 6.98 | 0.0433 |
| GO:0009051 | pentose-phosphate shunt, oxidative branch | 10 | 1 | 1.42 | 0.0098 |
| GO:0009817 | defense response to fungus, incompatible interaction | 4 | 0 | 0.57 | 0.0226 |
| GO:0009914 | hormone transport | 6 | 0 | 0.85 | 0.0416 |
| GO:0030029 | actin filament-based process | 20 | 1 | 2.85 | 0.0247 |
| GO:0031503 | protein complex localization | 34 | 4 | 4.84 | 0.0457 |
| GO:0032418 | lysosome localization | 2 | 0 | 0.28 | 0.0361 |
| GO:0032970 | regulation of actin filament-based process | 20 | 1 | 2.85 | 0.0247 |
| GO:0033499 | galactose catabolic process via UDP-galactose | 5 | 0 | 0.71 | 0.0049 |
| GO:0034629 | cellular protein complex localization | 6 | 0 | 0.85 | 0.0376 |
| GO:0035264 | multicellular organism growth | 4 | 1 | 0.57 | 0.0256 |
| GO:0035556 | intracellular signal transduction | 119 | 20 | 16.95 | 0.0121 |
| GO:0036265 | RNA (guanine-N7)-methylation | 4 | 0 | 0.57 | 0.0378 |
| GO:0042398 | cellular modified amino acid biosynthetic process | 13 | 1 | 1.85 | 0.0063 |
| GO:0043087 | regulation of GTPase activity | 5 | 0 | 0.71 | 0.0257 |
| GO:0043650 | dicarboxylic acid biosynthetic process | 14 | 1 | 1.99 | 0.0155 |
| GO:0044087 | regulation of cellular component biogenesis | 31 | 2 | 4.42 | 0.0256 |
| GO:0048868 | pollen tube development | 6 | 0 | 0.85 | 0.0192 |
| GO:0051128 | regulation of cellular component organization | 137 | 16 | 19.52 | 0.0198 |
| GO:0055114 | oxidation-reduction process | 89 | 13 | 12.68 | 0.0088 |
| GO:0060627 | regulation of vesicle-mediated transport | 20 | 0 | 2.85 | 0.0049 |
| GO:0065008 | regulation of biological quality | 136 | 11 | 19.37 | 0.0265 |
| GO:0072530 | purine-containing compound transmembrane transport | 5 | 0 | 0.71 | 0.0421 |
| GO:0097305 | response to alcohol | 10 | 1 | 1.42 | 0.0210 |
| GO:0097435 | supramolecular fiber organization | 18 | 1 | 2.56 | 0.0454 |
| GO:1901073 | glucosamine-containing compound biosynthetic process | 16 | 2 | 2.28 | 0.0243 |
| GO:1902074 | response to salt | 8 | 1 | 1.14 | 0.0094 |
| GO:1902903 | regulation of supramolecular fiber organization | 18 | 1 | 2.56 | 0.0454 |
