# Supplementary material for: Differential gene expression reveals mechanisms related to habitat divergence between hybridizing orchids from the Neotropical coastal plains
Source: BMC Plant Biol. 2020 Dec 10;20:554. doi: 10.1186/s12870-020-02757-x (PMC7731501; doi:10.1186/s12870-020-02757-x)
Supplement: Supplementary file 2 — Additional file 2: Appendix 1. Samples identity according to a Bayesian assignment analysis with microsatellites markers and flow cytometry. [file 12870_2020_2757_MOESM2_ESM.docx]

**APPENDIX 1**

**Samples determination**

*Molecular assignment test* - Although *E. fulgens* and *E. puniceoluteum* are well distinguished by their labellum colors and lobus size (Pinheiro and Barros 2006), the occurrence of natural hybrids in ICA and ICO makes the determination of each sampled specimen challenging. Thus, to confirm the identity of sampled individuals, we extracted genomic DNA from leaves following a modified CTAB protocol and genotyped nine nuclear microsatellite markers (Pinheiro et al. 2008a,b) employed in a previous study by Pinheiro et al. (2010). We then conducted a Bayesian analysis using the NewHybrids software (Anderson & Thompson 2002) to assign individuals of each hybrid zone (i.e. ICA and ICO) to each species or hybrids (as described in Pinheiro et al. 2010). For this, specimens from allopatric populations of *E. puniceoluteum* (PPR) and *E. fulgens* (TOR and BER) were used as a reference. Such analysis computes the posterior distribution of each sampled individual in belonging to one of the following categories: parental purebreds, hybrids F1, hybrids F2 and backcrosses. From 11 individuals sampled in ICA, three were determined as hybrids, while four out of 14 individuals from ICO were identified as hybrids (see Figure S1).


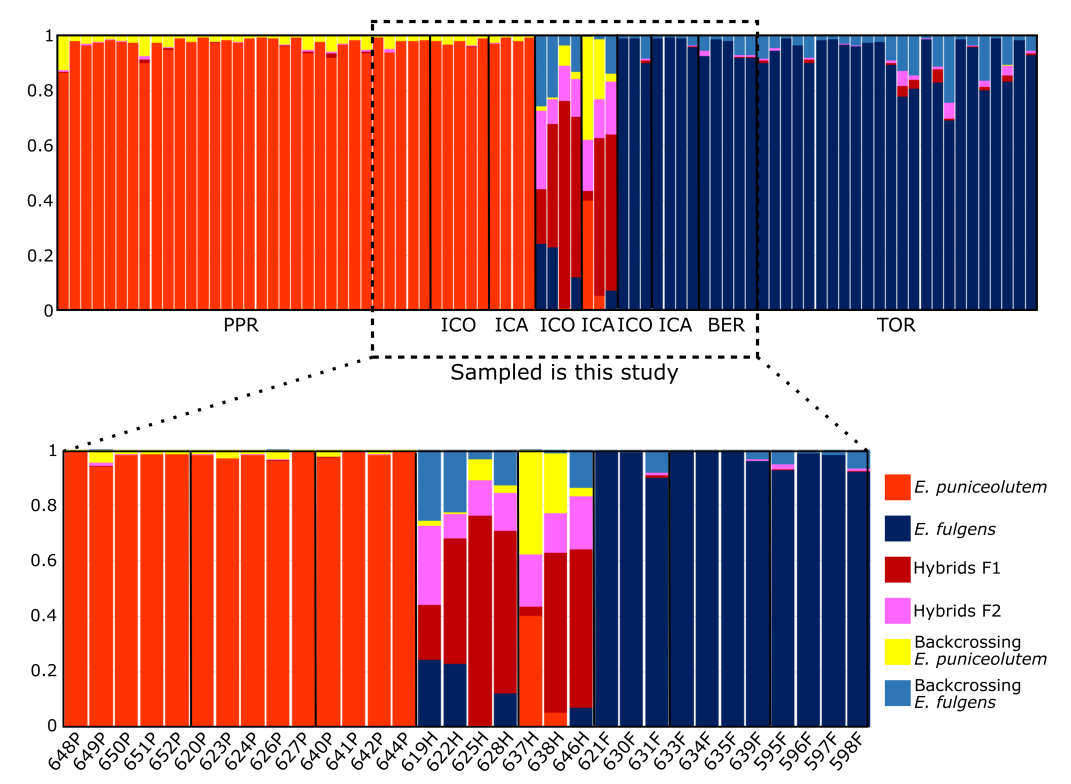


**Figure 1**. Probabilities of each individual from natural hybrid zones of Ilha Comprida (ICO) and Ilha do Cardoso (ICA) to be assigned as pure *Epidendrum fulgens*, pure *Epidendrum puniceoluteum,* F1 Hybrids, F2 Hybrids, or backcrossing hybrids, according to NewHybrids analysis. Additional samples from allopatric populations of *E. fulgens* from Torres (TOR) and *E. puniceoluteum* from Pontal do Paraná (PPR) was used as a reference of each species in this analysis (see Pinheiro et al. 2010).

*DNA content measurements* ***—*** Because *E. fulgens* and *E. puniceoluteum* have distinct chromosome numbers (2n=2x= 24 and 2n=4x= 52, respectively), we double-checked the identity of each sampled individual by estimating the DNA content levels using flow cytometry following Pinheiro et al. (2016). We prepared nuclear suspensions of each sample using *Ruscus aculeatus* fresh leaf tissue as an internal reference standard (2C = 20.59 pg; Vesely et al. 2012). We then performed the flow cytometry analysis on a BD Biosciences FACSCalibur flow cytometer (BD Biosciences, San Jose, CA, USA) and processed the data using CellQuestPro (Becton Dickinson, San Jose, CA, USA) and Flowing v. 2.5.1 softwares (www.flowingsoftware.com). The nuclear DNA content (sample peak mean divided by the standard peak mean) of each tested sample is shown in Table 1. As expected, individuals pointed as hybrids by the molecular assignment test have genome size ranging between the genomes size of parental species (i.e., Pg/2C=3.45 to 3.57 for hybrids, Pg/2C=2.84 to 2.91 for *E. fulgens*, and Pg/2C=4.65 to 4.80 for *E. puniceoluteum*; see Table 1).

**Table 1.** Nuclear DNA content of allopatric and sympatric individuals of *E. fulgens*, *E. puniceoluteum* and respective hybrids as estimated by flow cytometry.

| **Id** | **Species** | **Locality** | **Occurrence** | **Pg/2C** |
| --- | --- | --- | --- | --- |
| 648P | *E. puniceoluteum* | PPR | allopatric | 4.80 |
| 649P | *E. puniceoluteum* | PPR | allopatric | 4.74 |
| 650P | *E. puniceoluteum* | PPR | allopatric | 4.72 |
| 651P | *E. puniceoluteum* | PPR | allopatric | 4.70 |
| 652P | *E. puniceoluteum* | PPR | allopatric | 4.71 |
| 620P | *E. puniceoluteum* | ICO | sympatric | 4.73 |
| 623P | *E. puniceoluteum* | ICO | sympatric | 4.68 |
| 624P | *E. puniceoluteum* | ICO | sympatric | 4.78 |
| 626P | *E. puniceoluteum* | ICO | sympatric | 4.77 |
| 627P | *E. puniceoluteum* | ICO | sympatric | 4.72 |
| 640P | *E. puniceoluteum* | ICA | sympatric | 4.73 |
| 641P | *E. puniceoluteum* | ICA | sympatric | 4.65 |
| 642P | *E. puniceoluteum* | ICA | sympatric | 4.76 |
| 644P | *E. puniceoluteum* | ICA | sympatric | 4.69 |
| 619H | Hybrid | ICO | sympatric | 3.57 |
| 622H | Hybrid | ICO | sympatric | 3.45 |
| 625H | Hybrid | ICO | sympatric | 3.54 |
| 628H | Hybrid | ICO | sympatric | 3.81 |
| 637H | Hybrid | ICA | sympatric | 3.51 |
| 638H | Hybrid | ICA | sympatric | 3.50 |
| 646H | Hybrid | ICA | sympatric | 3.52 |
| 621F | *E. fulgens* | ICO | sympatric | 2.86 |
| 630F | *E. fulgens* | ICO | sympatric | 2.87 |
| 631F | *E. fulgens* | ICO | sympatric | 2.84 |
| 633F | *E. fulgens* | ICA | sympatric | 2.89 |
| 634F | *E. fulgens* | ICA | sympatric | 2.89 |
| 635F | *E. fulgens* | ICA | sympatric | 2.91 |
| 639F | *E. fulgens* | ICA | sympatric | 2.87 |
| 595F | *E. fulgens* | BER | allopatric | 2.88 |
| 596F | *E. fulgens* | BER | allopatric | 2.86 |
| 597F | *E. fulgens* | BER | allopatric | 2.90 |
| 598F | *E. fulgens* | BER | allopatric | 2.88 |

**References**

Anderson EC, Thompson EA (2002) A model-based method for identifying species hybrids using multilocus genetic data. Genetics, 160, 1217–1229.

Pinheiro F, Barros F (2006) *Epidendrum puniceoluteum*, uma nova espécie de Orchidaceae do litoral brasileiro. Hoehnea, 33, 247–250.

Pinheiro F., Santos, M. O., Barros, F., Meyer, D., Salatino, A., Souza, A. P., & Cozzolino, S. (2008). Isolation and characterization of microsatellite loci in the Brazilian orchid Epidendrum fulgens. Conservation genetics, 9(6), 1661-1663.

Pinheiro F., Santos, M. O., Palma‐Silva, C., Barros, F., Meyer, D., Salatino, A., ... & Cozzolino, S. (2008). Isolation and characterization of microsatellite loci in Epidendrum puniceoluteum, an endemic orchid from the Atlantic Rainforest. Molecular ecology resources, 8(5), 1114-1116.

Pinheiro F., de Barros F., Palma‐Silva C., Meyer D., Fay M. F., Suzuki R. M., ... & Cozzolino S. (2010). Hybridization and introgression across different ploidy levels in the Neotropical orchids Epidendrum fulgens and E. puniceoluteum (Orchidaceae). Molecular Ecology, 19(18), 3981-3994.

Pinheiro, F., Zanfra de Melo e Gouveia, T. M., Cozzolino, S., Cafasso, D., Cardoso‐Gustavson, P., Suzuki, R. M., & Palma‐Silva, C. (2016). Strong but permeable barriers to gene exchange between sister species of Epidendrum. American Journal of Botany, 103(8), 1472-1482.

Veselý, P., Bureš, P., Šmarda, P., & Pavlíček, T. (2011). Genome size and DNA base composition of geophytes: the mirror of phenology and ecology?. Annals of Botany, 109(1), 65-75.
